# Supplementary material for: Network topology of the gut microbiome associates with metabolic health in obesity
Source: Nat Commun. 2026 May 13;17:4113. doi: 10.1038/s41467-026-72588-1 (PMC13172010; doi:10.1038/s41467-026-72588-1)
Supplement: Supplementary file 1 — Supplementary Information [file 41467_2026_72588_MOESM1_ESM.pdf]

Supplementary Information

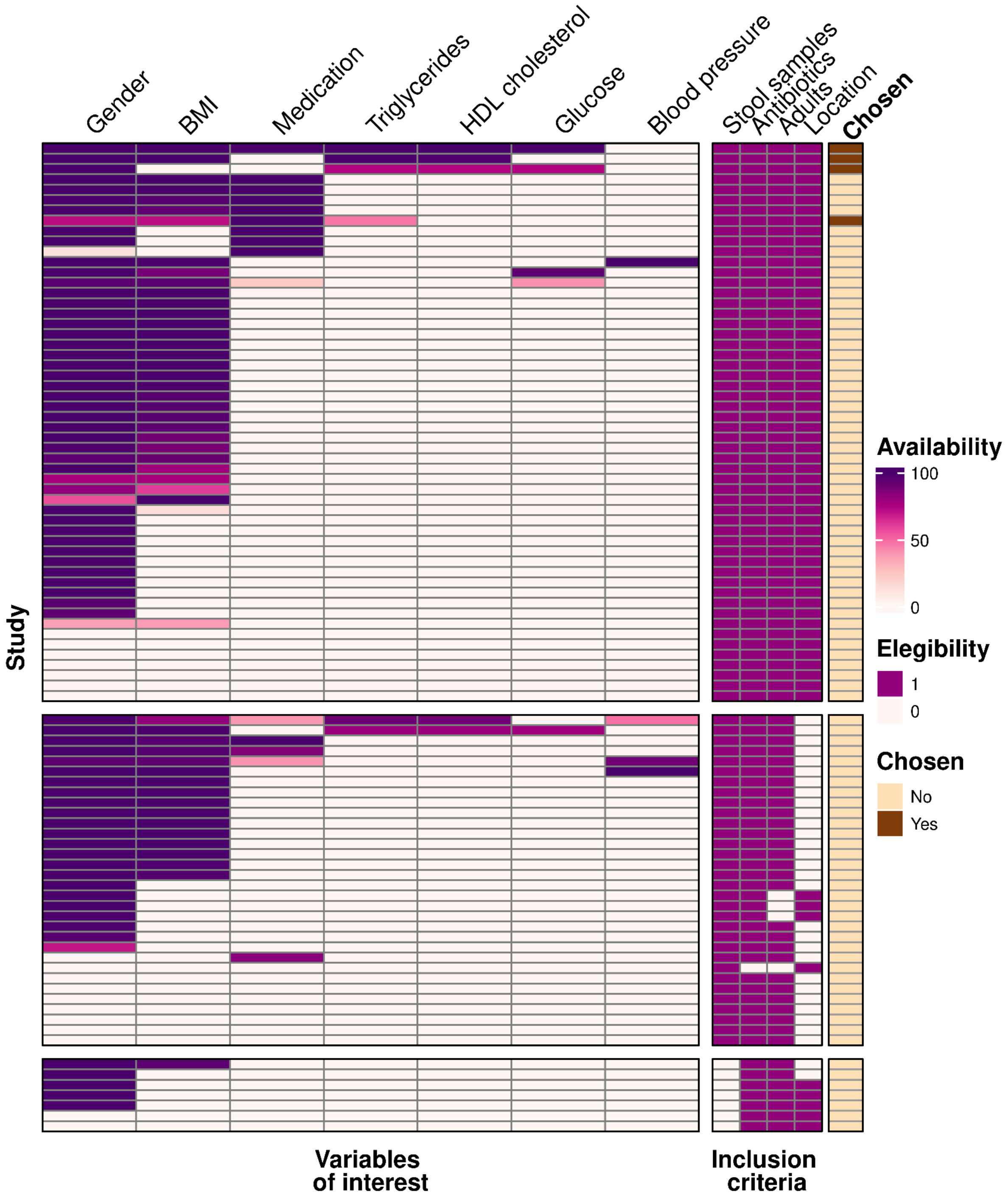

Supplementary Figure 1. curatedMetagenomicData study search. Heatmap showing availability of BMI and metabolic parameters among curatedMetagenomicData studies. The left-hand panel shows variable of interest availability in every study from curatedMetagenomicData as the percentage of non-missing values. This Figure reports data availability only on the curatedMetagenomicData R package. To enrich our dataset, source publications for the datasets were accessed to look for additional variables that might not have been included during the development of this resource (see Supplementary Figure 2). Inclusion criteria met by each study are shown: availability of feces samples, availability of samples from subjects without antibiotic intake, availability of samples from adult subjects, and location. Cells equal to 1 meet these criteria. Rows are separated into three panels based on eligibility criteria fulfillment. Studies chosen for subsequent analyses based on metadata availability are indicated. One of these studies was dropped due to unavailability of sequencing data (see Supplementary Figure 2).

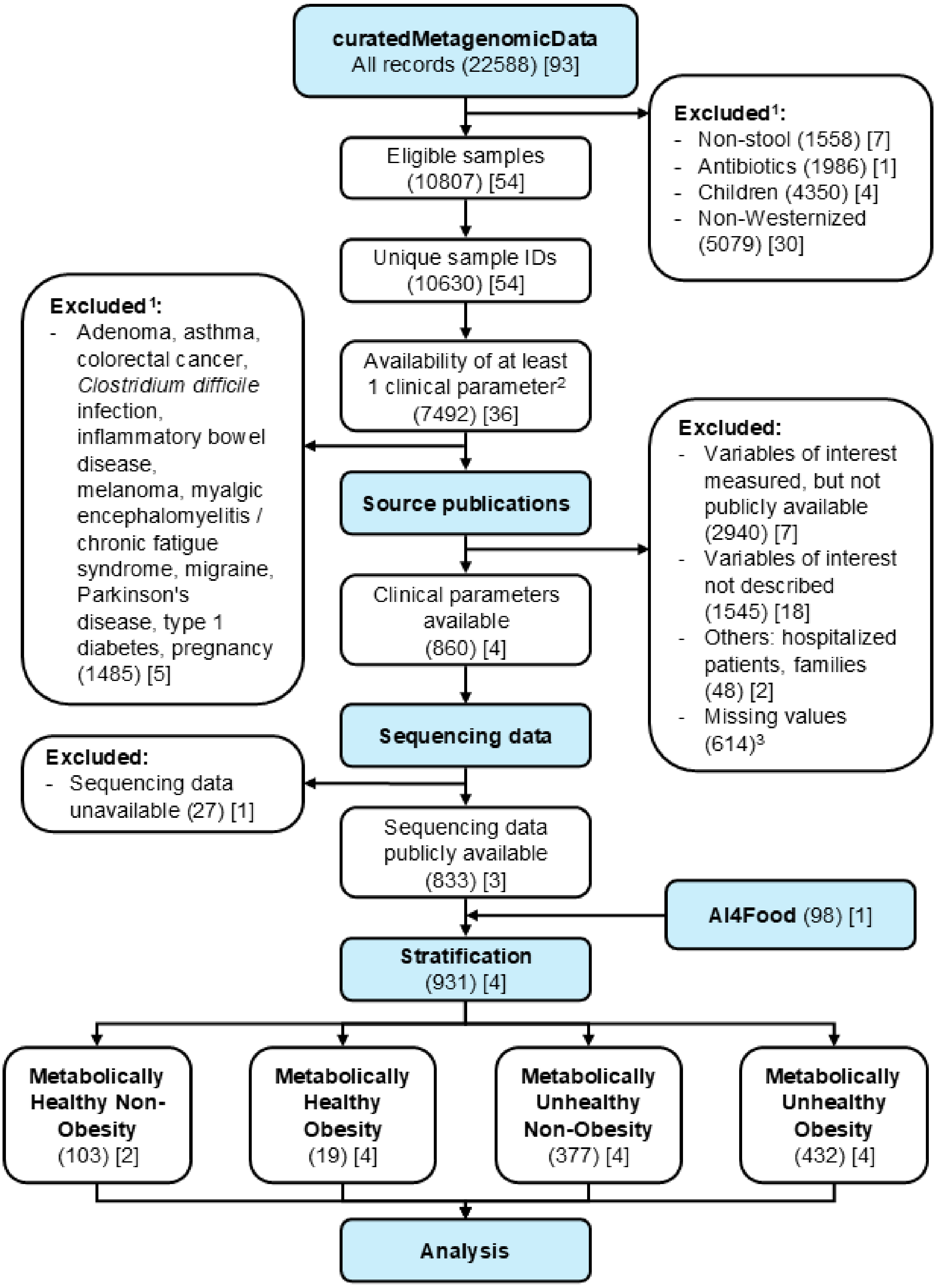

Supplementary Figure 2. Study selection process flow diagram. Sample sizes are given as (number of samples) [Number of studies where no samples could be included]. [1] Samples were excluded if any of these criteria were met. Some samples and studies met more than one exclusion criteria. [2] Clinical parameters considered: Medication intake, BMI, HDL cholesterol, systolic or diastolic blood pressure, triglycerides, glucose. [3] This includes 421 patients from MetaCardis_2020_a and 193 patients from HMP_2019_t2d.

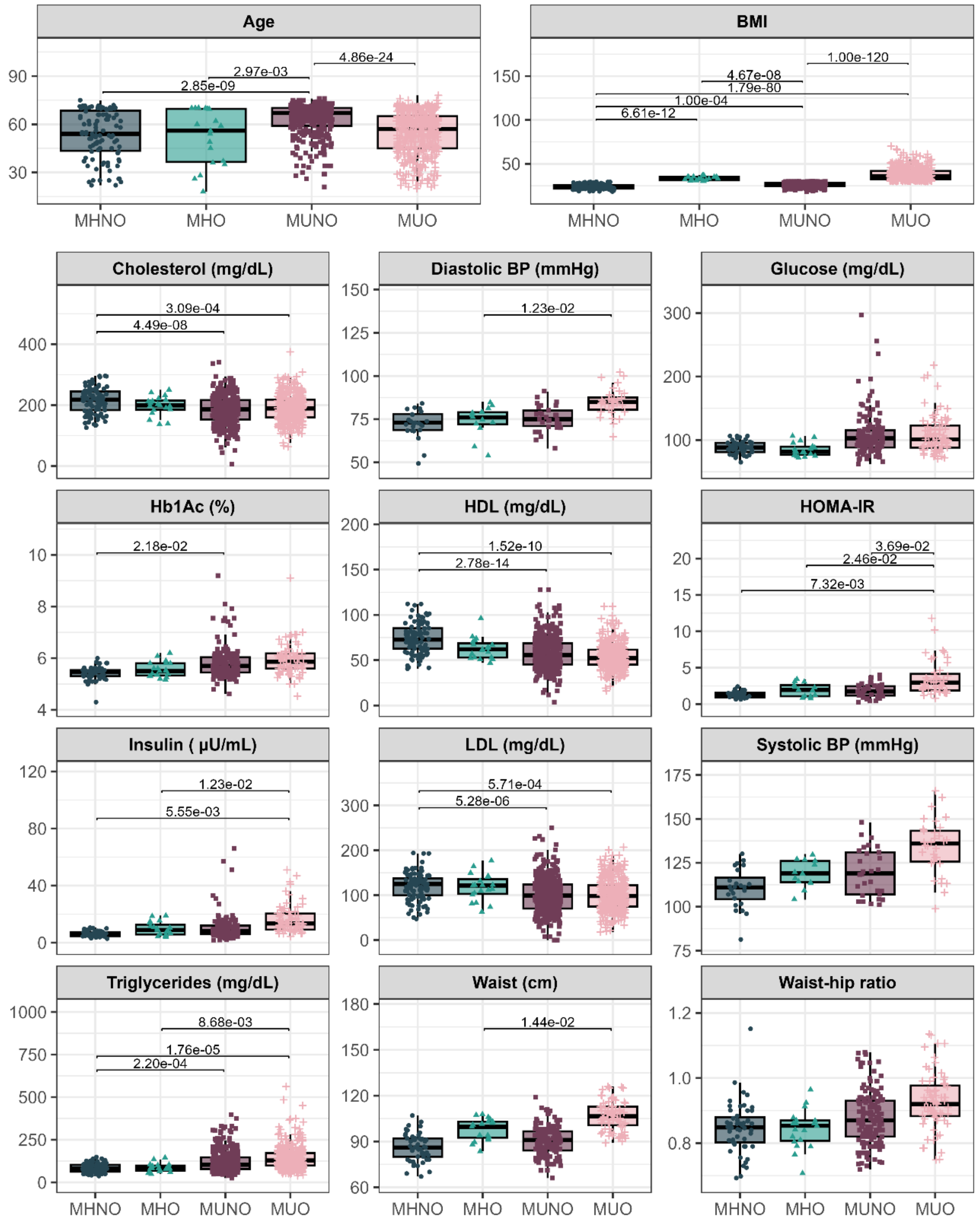

Supplementary Figure 3. Metadata exploration boxplots. Continuous variables with an adjusted p-value < 0.05 in Tables 2 and 3 were selected. Age, BMI: Dunn's Test for pairwise multiple comparisons, FDR (Benjamini-Hochberg) to adjust p-values for multiple comparisons. Remaining variables: estimated marginal means for post-hoc pairwise comparisons, Tukey's test for multiple comparisons. For detailed results, see Supplementary Data 1 and Supplementary Table 1. Sample sizes for each variable are given in Tables 2 and 3. BP: Blood pressure; HbA1c: Hemoglobin A1C; HOMA-IR: Homeostatic model assessment for insulin resistance; HDL: High-density lipoprotein; LDL: Low-density lipoprotein.

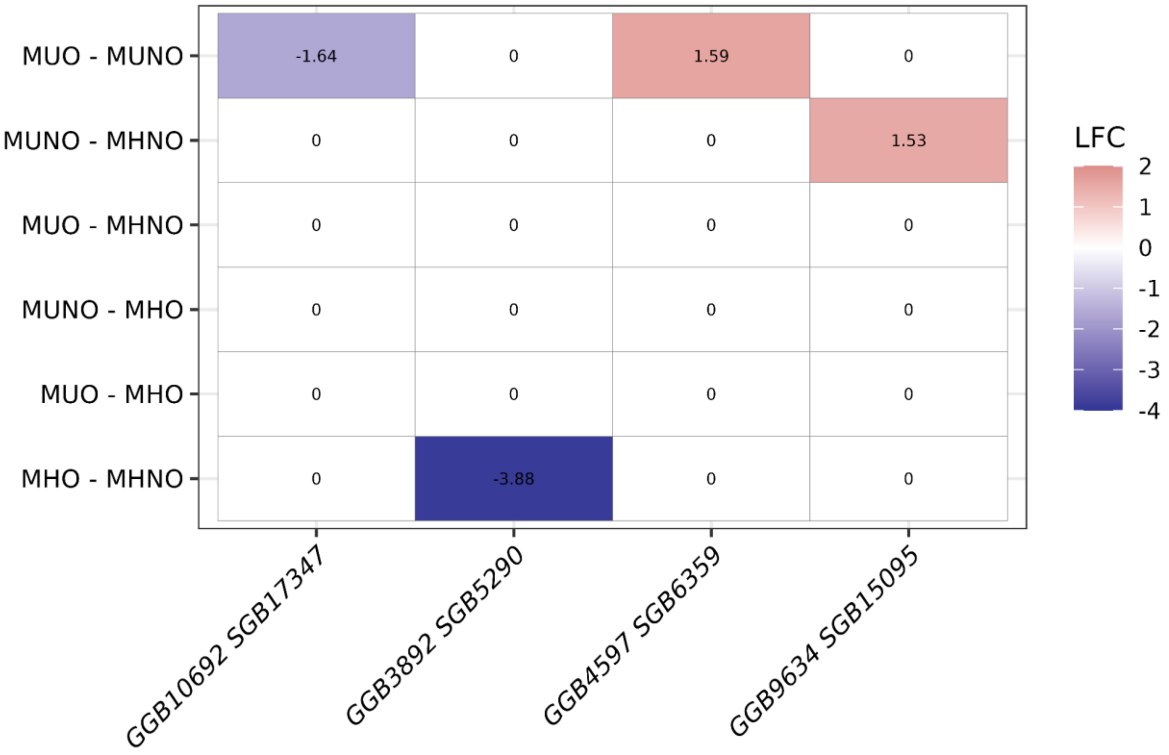

Supplementary Figure 4. Heatmap representing differential abundance analysis results (species-level). Differential abundances between MHNO (n = 103), MHO (n = 19), MUNO (n = 377) and MUO (n = 432) groups were calculated with ANCOM-BC2. Cells with values different from 0 represent comparisons with significant differences after false discovery rate (mdFDR) correction. Values and colors in the heatmap cells represent log fold-change (LFC) values.

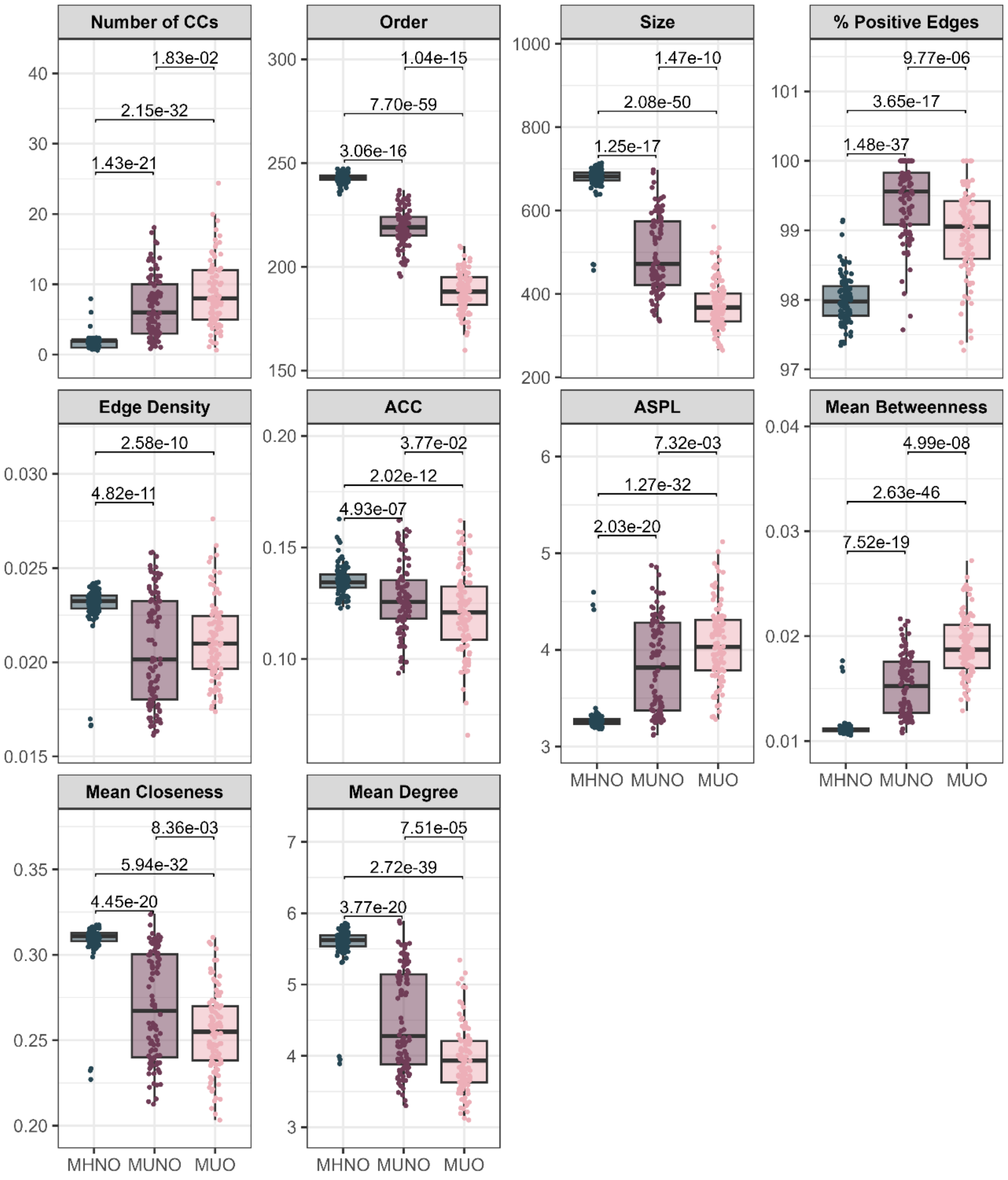

Supplementary Figure 5. Network validation analyses: Network metrics. MHNO, MUNO, and MUO individuals from all studies were randomly resampled to match the sample size of the MHNO group (n = 100) across 100 iterations. Boxplots represent network metrics from each resampled network. CCs: Connected components, ACC: Average clustering coefficient, ASPL: Average shortest path length. Differences between groups were tested with the Kruskal-Wallis rank-sum test and FDR-corrected. The two-sided Dunn's test followed by FDR correction were used for post-hoc comparisons.

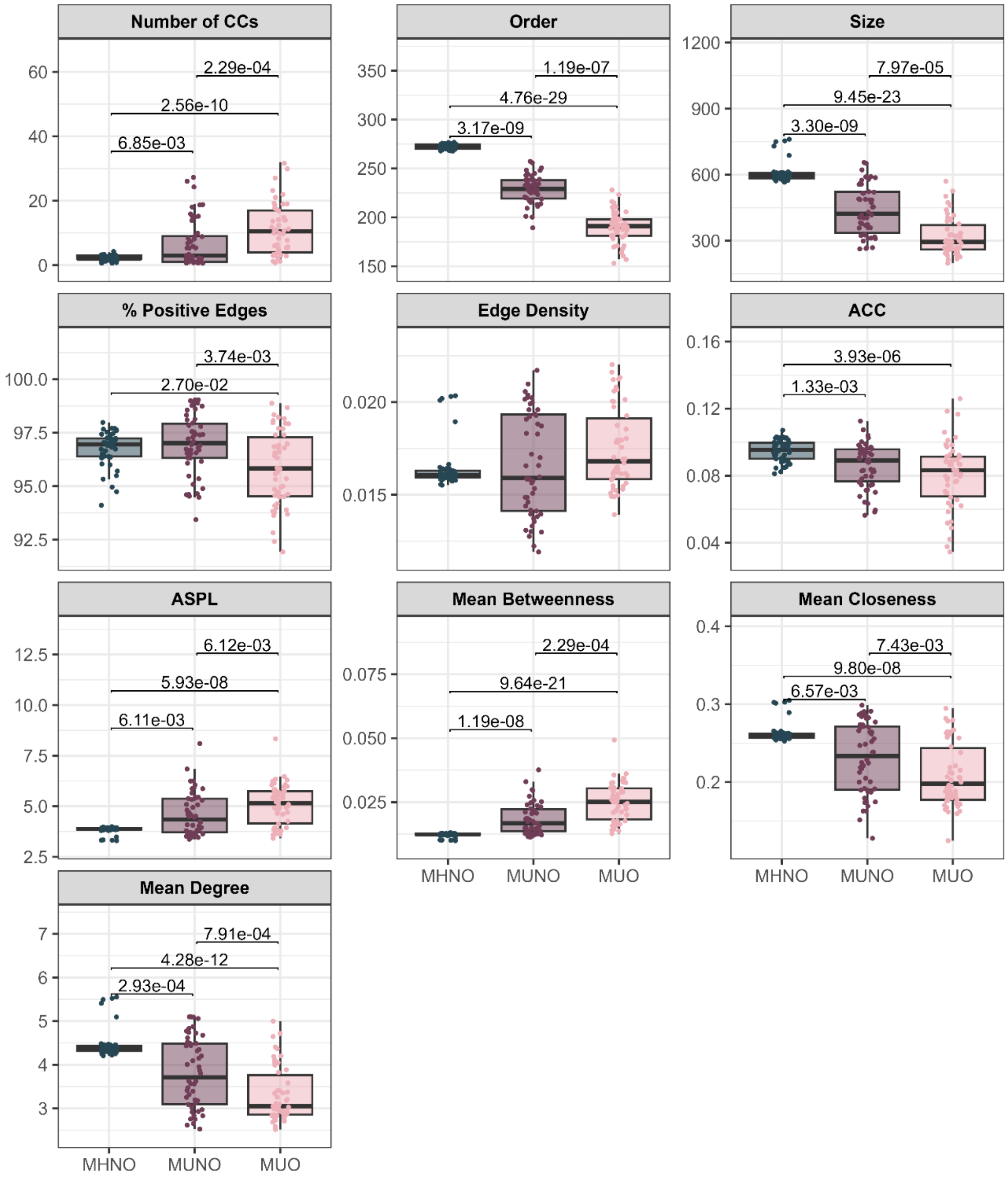

Supplementary Figure 6. Network validation analyses: Network metrics. MHNO, MUNO, and MUO individuals from the MetaCardis cohort were randomly resampled to match the sample size of the MHNO group (n = 50) across 50 iterations. Boxplots represent network metrics from each resampled network. CCs: Connected components, ACC: Average clustering coefficient, ASPL: Average shortest path length. Differences between groups were tested with the Kruskal-Wallis rank-sum test and FDR-corrected. The two-sided Dunn's test followed by FDR correction were used for post-hoc comparisons.

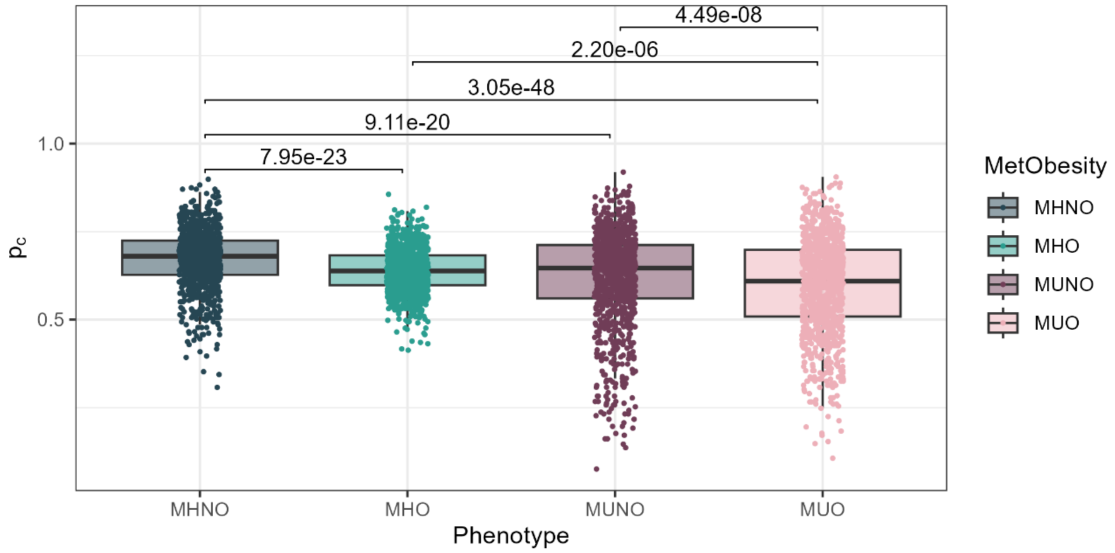

Supplementary Figure 7. Boxplots with percolation threshold p_c values obtained after n = 1000 random attack runs in each network. Differences between groups were tested with the Kruskal-Wallis rank-sum test. Two-sided Dunn's test followed by FDR correction was used for post-hoc comparisons

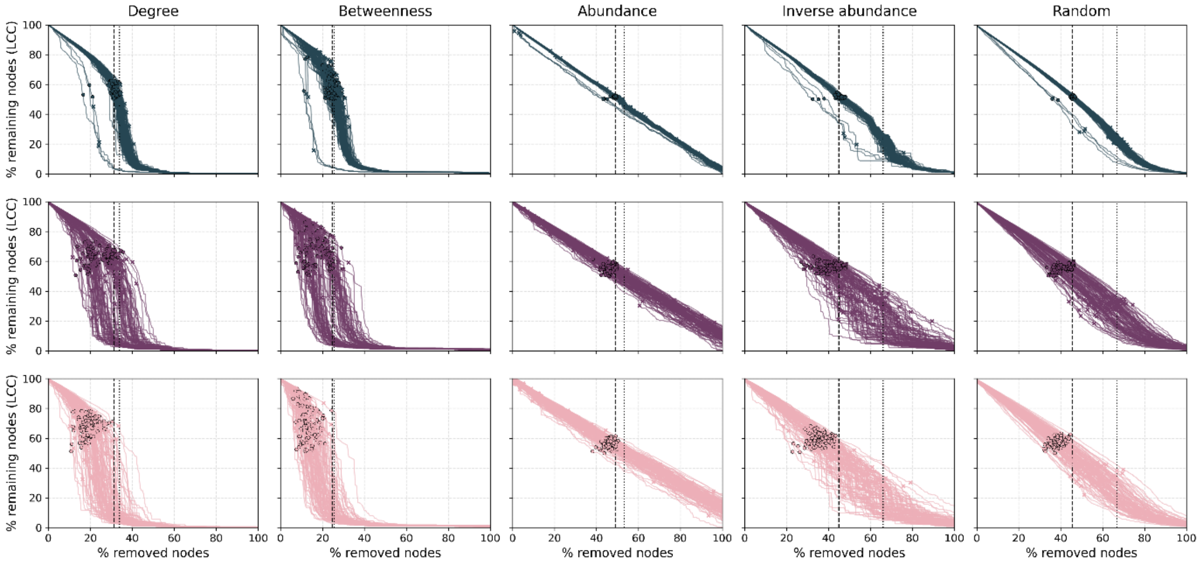

Supplementary Figure 8. Network validation analyses: Network stability. MHNO (top row), MUNO (center row), and MUO (bottom row) individuals from all studies were randomly resampled to match the sample size of the MHNO group (n = 100) across 100 iterations. LCC decay curves, together with their NR_{50} (circles) and p_c (triangles) values, are reported for attacks based on node degree (**a**), betweenness centrality (**b**), increasing mean relative abundance (**c**), decreasing mean relative abundance (**d**), and random (10 iterations) (**e**). Vertical lines represent the mean NR_{50} (dashed line) and p_c (dotted line) values from MHNO networks.

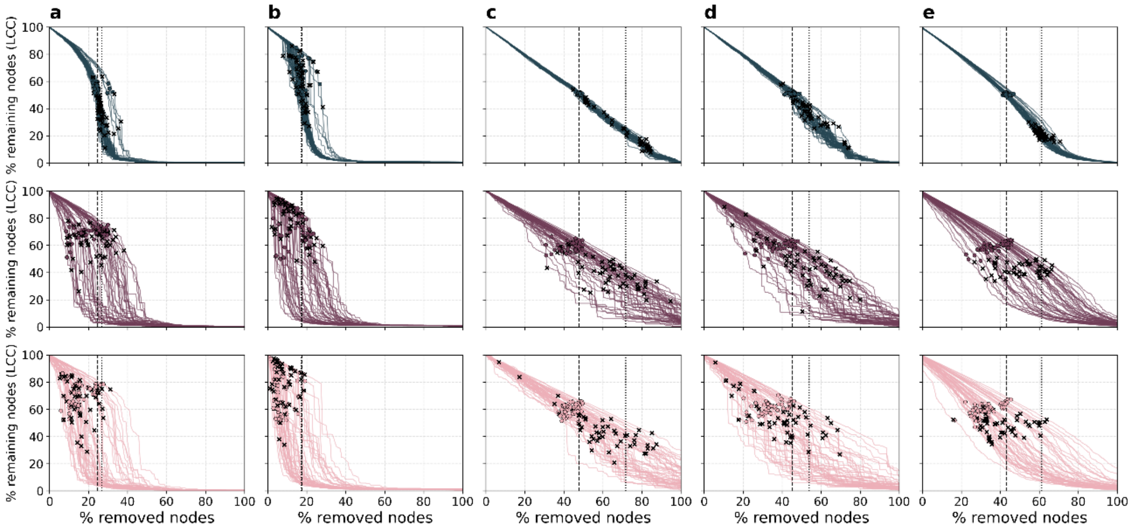

Supplementary Figure 9. Network validation analyses: Network stability. MHNO (top row), MUNO (center row), and MUO (bottom row) individuals from the MetaCardis cohort were randomly resampled to match the sample size of the MHNO group (n = 50) across 50 iterations. LCC decay curves, together with their NR_{50} (circles) and p_c (triangles) values, are reported for attacks based on node degree (**a**), betweenness centrality (**b**), increasing mean relative abundance (**c**), decreasing mean relative abundance (**d**), and random (10 iterations) (**e**). Vertical lines represent the mean NR_{50} (dashed line) and p_c (dotted line) values from MHNO networks.

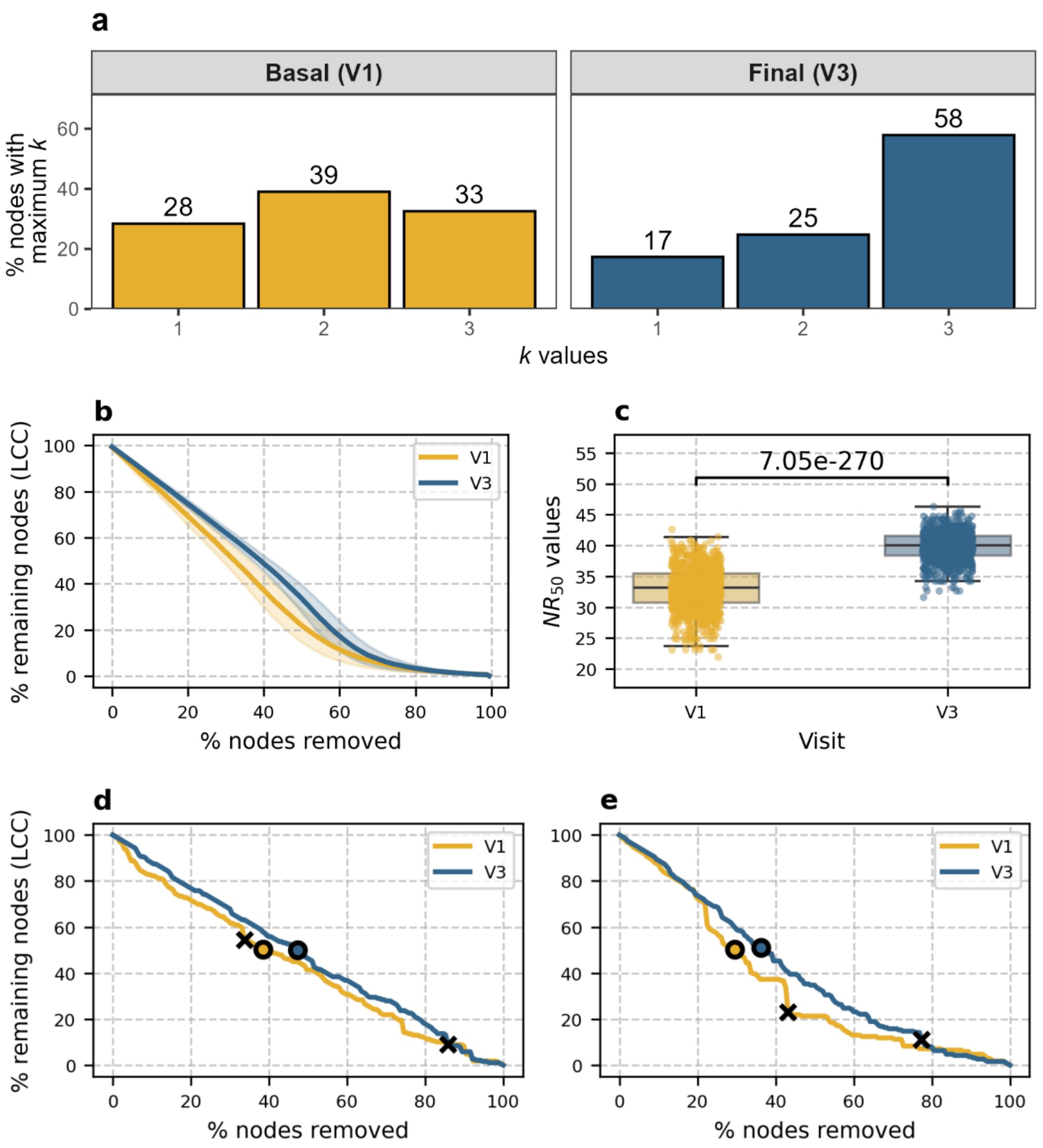

Supplementary Figure 10. Nutritional intervention networks. Network analyses performed on AI4Food networks before and after the AI4Food nutritional intervention. **a)** *K*-core distribution histograms. **b)** Mean curve and standard deviation after random attacks (m = 1000 iterations). **c)** Histogram of NR_{50} values obtained from (b). **d-e)** LCC decay curve representing attacks based on decreasing (**d**) or increasing (**e**) mean abundances.

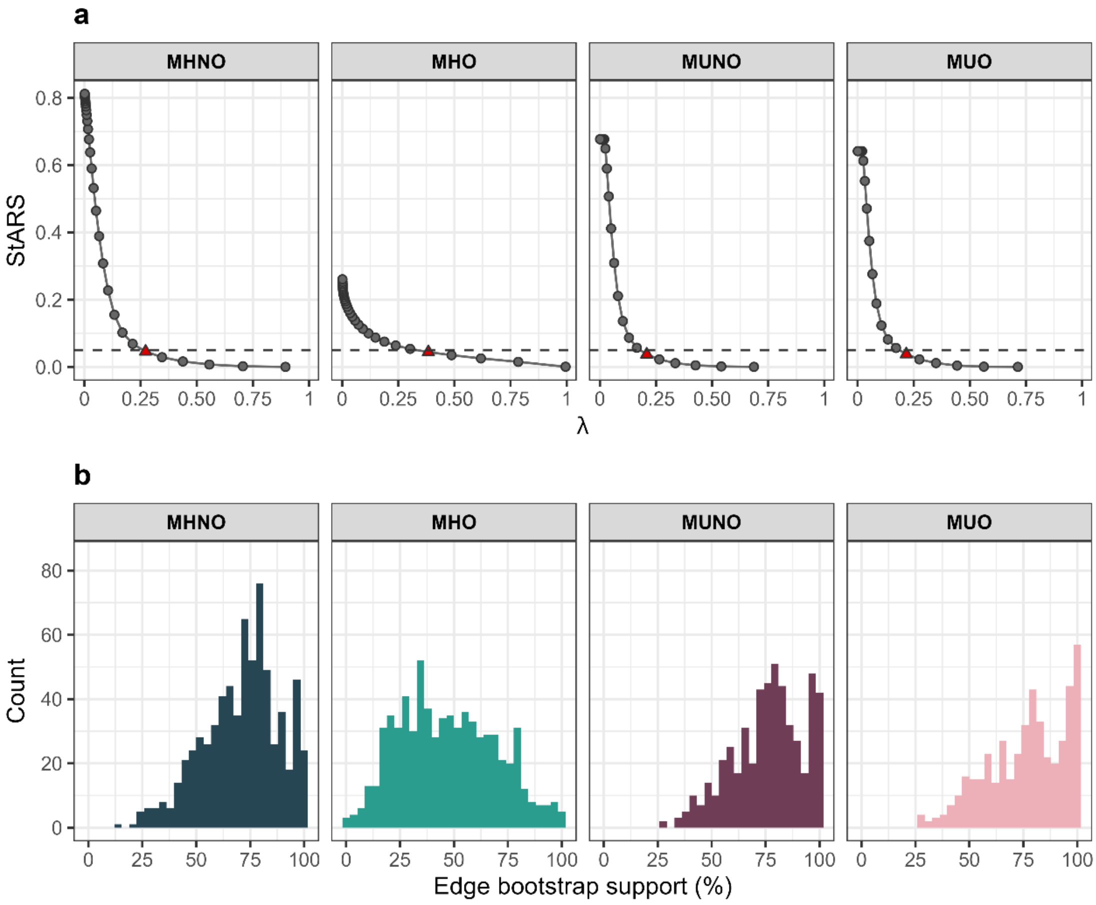

Supplementary Figure 11. Network construction support. Networks for each phenotype were built with SPIEC-EASI using the Meinshausen-Bühlmann neighborhood selection method and StARS-based model selection. **a)** StARs stability curves. The optimal λ value chosen for network construction is shown in a different shape (triangle) and color. **b)** Edge bootstrap support histograms.

Supplementary Tables

Supplementary Table 1. Metadata exploration: group-wise comparisons. Age and BMI. Dunn's test for pairwise multiple comparisons was performed as a post-hoc test following the Kruskal-Wallis test on Table 2. Multiple corrections were controlled using Benjamini-Hochberg's FDR correction.

Feature	Comparison	z-score	p-value	p-value (adjusted)
Age (years)	MHNO - MHO	-0.27	0.79	0.92
	MHNO - MUNO	6.12	9.50×10^{-10}	2.85×10^{-9}
	MHNO - MUO	-0.41	0.68	0.92
	MHO - MUNO	3.18	0.001	0.003
	MHO - MUO	0.10	0.93	0.92
	MUNO - MUO	-10.29	8.10×10^{-25}	4.86×10^{-24}
BMI (kg/m^2)	MHNO - MHO	6.96	3.31×10^{-12}	6.61×10^{-12}
	MHNO - MUNO	3.93	8.36×10^{-5}	1.00×10^{-4}
	MHNO - MUO	19.06	6.00×10^{-81}	1.79×10^{-80}
	MHO - MUNO	-5.54	3.11×10^{-8}	4.67×10^{-8}
	MHO - MUO	1.50	0.14	0.13
	MUNO - MUO	23.44	1.67×10^{-121}	1.00×10^{-120}

Supplementary Table 2. NR_{50} and p_c values for all attack frameworks shown in Figure 5.

	**Node removal 50 NR_{50}**	**Critical threshold p_c**
**Degree**		
MHNO	31.98	34.41
MHO	31.00	35.06
MUNO	22.22	22.73
MUO	21.30	23.67
**Betweenness**		
MHNO	24.70	25.10
MHO	24.00	24.35
MUNO	17.17	17.68
MUO	15.38	8.88
**Abundance (decreasing)**		
MHNO	48.99	51.82
MHO	48.71	87.08
MUNO	47.47	46.97
MUO	21.30	62.13
**Abundance (increasing)**		
MHNO	44.53	67.21
MHO	46.49	73.06
MUNO	40.91	64.14
MUO	40.83	81.07

Supplementary Table 3. Network metrics before and after the AI4Food nutritional intervention. Properties except order and size are given for the largest connected component.

	Basal	Final	p[1]
**Order**	183	196	
**Size**	271	357	
**% negative edges**	2.95	7.28	
**Edge density**	1.91×10^{-2}	1.99×10^{-2}	
**Number of CCs[2]**	15	7	
**Degree (mean)**	3.21	3.76	8.61×10^{-3}
**Shortest path length (mean)**	5.09	4.07	$<2.2 \times 10^{-16}$
**Betweenness centrality (mean)**	2.72×10^{-2}	1.85×10^{-2}	6.86×10^{-2}
**Closeness centrality (mean)**	0.20	0.25	1.21×10^{-24}

[1] Wilcoxon rank sum test comparing node distributions across the four networks. Values are FDR-corrected (Benjamini-Hochberg). [2] CCs: Connected components.

Supplementary Table 4. Summary of included studies and their sequence accession numbers.

	**AI4Food**[1]	**FengQ_2015**[2]	**KarlssonFH_2013**[3]	**MetaCardis_2020_a**[4]
**Geography**	Spain	Austria	Sweden	France (n = 383), Germany (n = 246)
**Age (years)**[5]	51 (38, 60)	68 (65, 71)	70 (70, 71)	58 (47, 65)
**Sex (% female)**	69%	41%	100%	49%
**DNA extraction method**	QIAamp Fast DNA Stool Mini Kit	Unreported	Unreported	International Human Microbiome Standards (IHMS) guidelines (SOP 07 V2 H)
**Sequencing method**	Illumina NovaSeq 6000	Illumina HiSeq 2000	Illumina HiSeq 2000	Ion Torrent Proton
**Sequencing depth**[5,6]	22.5 (20.6, 26.1)	26.1 (23.4, 29.3)	27.8 (21.7, 38.9)	8.9 (5.5, 12.0)
**Average read length**[5]	150 (150, 150)	93 (89, 95)	101 (101, 101)	141 (136, 145)

1 PRJEB87701. **2** PRJEB7774. **3** PRJEB1786. **4** PRJEB41311; PRJEB38742; PRJEB37249. **5** Median (Q1, Q3). **6** Number of paired reads for paired-end datasets and number of reads for single-end datasets (millions).

Supplementary Methods

Phenotype assignment

AI4Food. This cohort had all relevant metadata to apply the BioSHaRE-EU classification criteria. For improved transparency, we have included scripts for subject classification using metadata from the study's GitHub repository (https://github.com/AI4Food/AI4FoodDB).

Karlsson. Supplementary Table 3 from the source publication[1] contains information on BMI; HDL cholesterol, glucose and triglyceride plasma values; antidiabetics or statins intake; and glucose tolerance (75-g standardized oral glucose tolerance test), assigning subjects as having normal glucose tolerance (NGT), impaired glucose tolerance (IGT) or type 2 diabetes (T2D). All subjects assigned to MHNO (n = 22) or MHO (n = 6) labels fit 3 out of 4 BioSHaRE-EU conditions regarding clinical parameters, only lacking information on systolic and diastolic blood pressure. Subjects with IGT or T2D were automatically assigned to MUNO/MUO groups, since alterations in glucose homeostasis are a hallmark of MU phenotypes.

Feng. Since this study focuses on colorectal cancer and adenoma, the first step was to exclude such subjects so that only healthy controls remained. Then, we accessed the following information from Supplementary Table 1 in the source publication[2]: BMI; diagnosis of diabetes, hypertension, or metabolic syndrome as defined by the National Cholesterol Education Program Adult Treatment Panel[3]; fasting plasma glucose, triglycerides, and HDL cholesterol concentrations; and other clinical variables including fatty liver ultrasound detection. In this cohort, all subjects assigned to the MHNO category (n =6) fit 3 out of the 4 BioSHaRE-EU criteria[4], only lacking specific systolic and diastolic pressure values. Nevertheless, none of them are hypertense. Moreover, since this cohort has available data on hepatic steatosis, which is a hallmark of MH>MU transition, we have ensured that none of the subjects assigned to the MHNO phenotype has this condition. Paired-end reads were used for subsequent analyses.

MetaCardis. We downloaded Supplementary Tables 1a and 1b from the dataset's source publication[5] to retrieve information regarding the MetaCardis study groups. We have also accessed Zenodo record with doi 10.5281/zenodo.4674360 to gather information on antibiotic and other drugs intake (*antibiotics_20201210.r* and *cmd_drugs_20201210.r* files from folder *metadata.tar.gz*); sex, age, BMI and geographic location (*demographic_20201210.r* file from *metadata.tar.gz*); lipidic panel including total

plasma triglycerides, total plasma cholesterol, and HDL and LDL cholesterol (file *hub.lipo.v3.data.frame.r* from *input_features.tar.gz*).

First, we confirmed that none of the included subjects were taking antibiotics and to assign obesity and non-obesity labels. To further classify subjects as part of the MHNO, MUNO or MUO groups, we assigned the MHNO label to subjects belonging to MetaCardis group 8, defined by the absence of coronary artery disease, type 2 diabetes or metabolic syndrome as defined by the International Diabetes Federation[6]. Their plasma HDL and triglyceride concentrations fit the BioSHaRE-EU criteria for metabolic health. Individuals from MetaCardis group 8 with missing values for such concentrations were discarded. Finally, none of these subjects were under treatment for any of the drugs considered in the study. This is a comprehensive list including acarbose, angiotensin-converting-enzyme inhibitors, angiotensin receptor blockers, centrally acting antihypertensive drugs, amiodarone, renin antagonists, anti-thrombocytes, aspirin, beta-blockers, insulin bolus, calcium antagonists, plavix, anticoagulants, digitalis, diuretics, ezetimibe, fibrate, glitazone, glucagon-like peptide-1 agonists, insulin, K-sparing diuretic, metformin, nitrate, proton pump inhibitors or related drugs, red rice, sodium-glucose cotransporter-2 inhibitors, statins, sulfonylurea, thiazidique, gout drugs, antiarrhythmics, or heparine.

We acknowledge that fasting glucose and blood pressure values are missing for this cohort. However, since all subjects belong to MetaCardis group 8, we know that none of them present metabolic syndrome as defined by the International Diabetes Federation. Therefore, we are confident that the 51 individuals labelled as MHNO are representative of a metabolically healthy phenotype within the limitations of publicly available data.

As for individuals with MUNO and MUO, subjects assigned to these groups belong to MetaCardis groups different than group 8, or are positive for antihypertensive, antilipidemic or antibiotic drug intake, or have HDL or triglyceride concentrations outside the healthy BioSHaRE-EU criteria[4].

Confounder analysis: Neighborhood selection with covariates

To validate whether differences in age, sex and BMI among the groups defined in our study were having an influence on network connectivity and topology, we performed an additional analysis based on neighborhood selection with covariates. Such methods allow to include additional variables as part of the input table used to infer the microbial co-occurrence matrix. FlashWeave[7] (FW) is one of such methods. This tool allows the detection of edges potentially influenced by metadata items such as, in our case, BMI,

age or sex. To test for the potential effect these variables might be having on network topology, we ran FW with and without them. This was done on MUNO (n = 377) and MUO (n = 432) groups, since they have sufficiently large sample sizes for stable FW-based network inference. FlashWeave version 0.19.2 was ran on Julia version 1.11.6.

FW networks were mostly unaffected by metadata inclusion. In the MUNO network, 18/213 taxa were connected to one of the variables, representing 8.45% of the total microbial nodes. Out of the 721 edges in the microbe+confounder network, 26 (3.61%) of them were influenced by metadata, with 19 of them representing microbe-metadata associations and 7 of them representing microbe-microbe associations that could be influenced by metadata nodes. As for the MUO case, 10/190 taxa (5.26%) were connected to metadata variables, with 12/623 edges (1.93%) being influenced by them (10 taxa-metadata, 1 taxa-taxa and 1 metadata-metadata edges). None of the metadata nodes were keystone taxa according to our criteria (betweenness and degree 90th percentile). Figure S1 represents metadata nodes and their first neighbors in both MUNO and MUO cases.

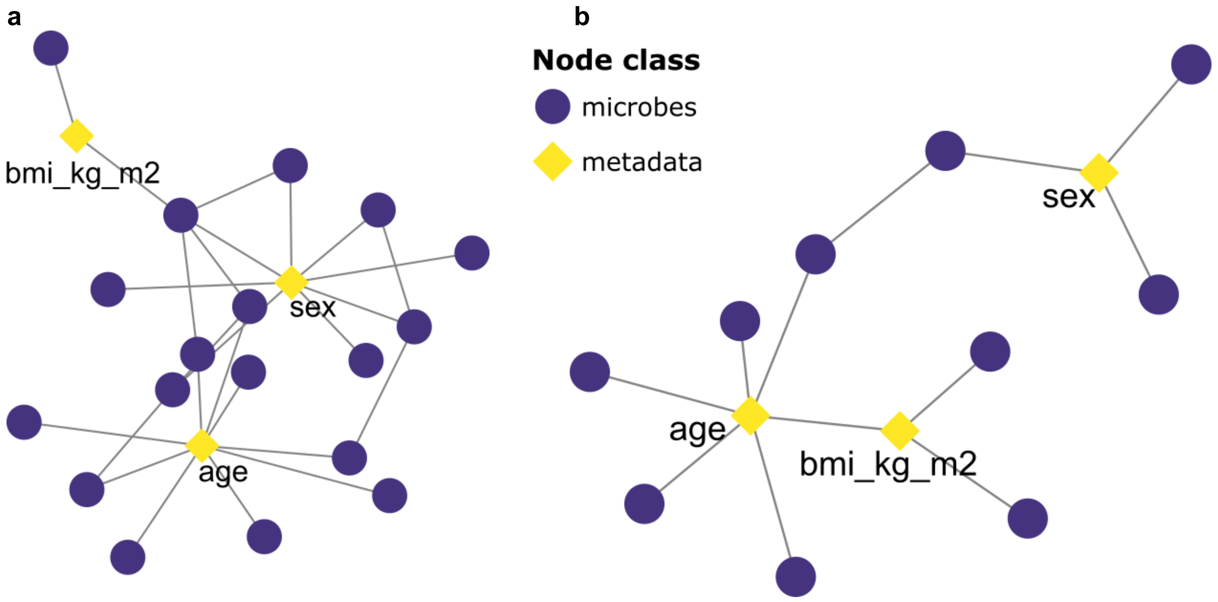

Supplementary Figure 12. Metadata influence on FlashWeave networks. Cytoscape visualization of metadata nodes and their first neighbors in MUNO **(a)** and MUO **(b)** groups.

We also compared the amount of edges present in the microbial FW network that were lost after metadata inclusion. Few edges were lost after including age, sex and BMI (15/710 edges in MUNO and 1/613 edges in the MUO case), as seen in Figure S2.

a b

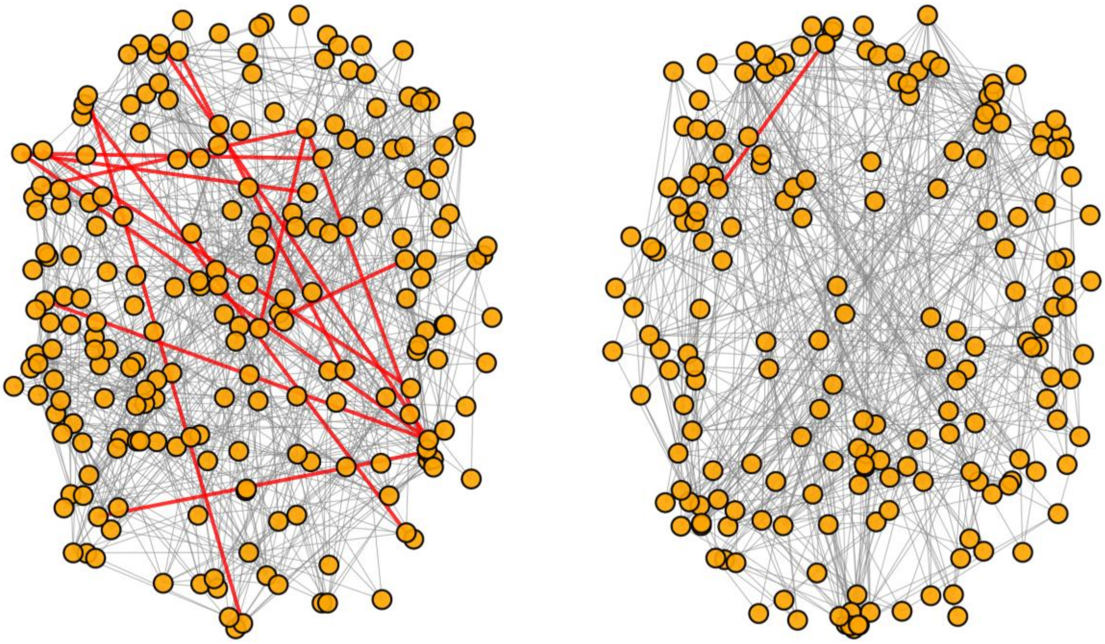

Supplementary Figure 13. NetworkX visualization of FlashWeave networks. Networks in the image are composed exclusively of microbial nodes. Highlighted edges represent microbe-microbe edges that were lost when age, sex and BMI were included in the FW input matrix. **A)** MUNO, **B)** MUO.

Supplementary References

1. Karlsson, F. H. *et al.* Gut metagenome in European women with normal, impaired and diabetic glucose control. *Nature* **498**, 99–103 (2013).
2. Feng, Q. *et al.* Gut microbiome development along the colorectal adenoma–carcinoma sequence. *Nat Commun* **6**, 6528 (2015).
3. Expert Panel on Detection and Treatment of High Blood Cholesterol in Adults, E. Executive Summary of The Third Report of The National Cholesterol Education Program (NCEP) Expert Panel on Detection, Evaluation, And Treatment of High Blood Cholesterol In Adults (Adult Treatment Panel III). *J. Am. Med. Assoc.* **285**, 2486–2497 (2001).
4. van Vliet-Ostaptchouk, J. V. *et al.* The prevalence of metabolic syndrome and metabolically healthy obesity in Europe: a collaborative analysis of ten large cohort studies. *BMC Endocr Disord* **14**, 9 (2014).
5. Forslund, S. K. *et al.* Combinatorial, additive and dose-dependent drug-microbiome associations. *Nature* **600**, 500–505 (2021).
6. Alberti, K. G. M. M., Zimmet, P. & Shaw, J. The metabolic syndrome–a new worldwide definition. *Lancet* **366**, 1059–1062 (2005).
7. Tackmann, J., Rodrigues, J. F. M. & Mering, C. von. Rapid Inference of Direct Interactions in Large-Scale Ecological Networks from Heterogeneous Microbial Sequencing Data. *Cell Syst.* **9**, 286-296.e8 (2019).
