# Supplementary material for: Network topology of the gut microbiome associates with metabolic health in obesity
Source: Nat Commun. 2026 May 13;17:4113. doi: 10.1038/s41467-026-72588-1 (PMC13172010; doi:10.1038/s41467-026-72588-1)
Supplement: Supplementary file 2 — Description of Additional Supplementary Files [file 41467_2026_72588_MOESM2_ESM.pdf]

Supplementary Data 1. Metadata exploration: group-wise comparisons. Anthropometrical and biochemical parameters. Variables showing an FDR-adjusted $p < 0.05$ in Table 2 were selected for pairwise contrasts using estimated marginal means (two-sided). Tukey's method was used to control for multiple comparisons. For a visual representation, see Supplementary Figure 3.

Supplementary Data 2. STORMS checklist.

Supplementary Data 3. Subject IDs, phenotype classifications and accession numbers for all samples included in this study.
