# Supplementary material for: Network topology of the gut microbiome associates with metabolic health in obesity
Source: Nat Commun. 2026 May 13;17:4113. doi: 10.1038/s41467-026-72588-1 (PMC13172010; doi:10.1038/s41467-026-72588-1)
Supplement: Supplementary file 6 — Reporting Summary [file 41467_2026_72588_MOESM6_ESM.pdf]

nature portfolio

Corresponding author(s): Laura Judith Marcos-Zambrano, Enrique Carrillo de Santa Pau

Last updated by author(s): Apr 14, 2026

Reporting Summary

Nature Portfolio wishes to improve the reproducibility of the work that we publish. This form provides structure for consistency and transparency in reporting. For further information on Nature Portfolio policies, see our Editorial Policies and the Editorial Policy Checklist.

Statistics

For all statistical analyses, confirm that the following items are present in the figure legend, table legend, main text, or Methods section.

n/a	Confirmed	
☐	☒	The exact sample size (*n*) for each experimental group/condition, given as a discrete number and unit of measurement
☐	☒	A statement on whether measurements were taken from distinct samples or whether the same sample was measured repeatedly
☐	☒	The statistical test(s) used AND whether they are one- or two-sided<br>*Only common tests should be described solely by name; describe more complex techniques in the Methods section.*
☐	☒	A description of all covariates tested
☐	☒	A description of any assumptions or corrections, such as tests of normality and adjustment for multiple comparisons
☐	☒	A full description of the statistical parameters including central tendency (e.g. means) or other basic estimates (e.g. regression coefficient) AND variation (e.g. standard deviation) or associated estimates of uncertainty (e.g. confidence intervals)
☐	☒	For null hypothesis testing, the test statistic (e.g. *F*, *t*, *r*) with confidence intervals, effect sizes, degrees of freedom and *P* value noted<br>*Give P values as exact values whenever suitable.*
☒	☐	For Bayesian analysis, information on the choice of priors and Markov chain Monte Carlo settings
☒	☐	For hierarchical and complex designs, identification of the appropriate level for tests and full reporting of outcomes
☐	☒	Estimates of effect sizes (e.g. Cohen's *d*, Pearson's *r*), indicating how they were calculated

Our web collection on statistics for biologists contains articles on many of the points above.

Software and code

Policy information about availability of computer code

Data collection	Publicly available data were obtained from the curatedMetagenomicData R package (version 3.8), the European Nucleotide Archive, and source publications from each study.<br>Stool samples from the AI4Food project were collected and frozen at -80 ºC. DNA isolation was performed using the QIAamp Fast DNA Stool Mini Kit DNA extraction following the manufacturer's instructions (QIAGEN, Hilden, Germany). Microbial analysis was performed by metagenomics shotgun sequencing on the NovaSeq 6000 Illumina platform (2 x 150 bp) with a coverage of approximately 6 GB per sample, equivalent to ~40 million reads.
Data analysis	Host read removal (hg38 version) and quality control consisting of adapter auto-detection, per-base quality trimming (Q≥20), removal of reads shorter than 50 bp, and filtering of reads with >5% ambiguous bases were performed with fastp (v0.25.0). The taxonomic profiling and quantification of relative abundances have been performed using MetaPhlAn4 (version 4.2.2) mapping against the mpa_vJan25_CHOCOPhlAnSGB_202503 database.<br>The MMUPHin R package (version 1.14) was used for batch effect correction caused by the study of origin while controlling for the effect of metabolic health and obesity. R packages vegan (version 2.6-8), microbiome (version 1.22) and phyloseq (version 1.46) were also used in microbiome analyses. Differential abundance testing was performed with ANCOM-BC2 (version 2.2.2) R package.<br>Network analyses: SpiecEasi R package (version 1.1.3), igraph R package (version 2.1.4), NetworkX Python library (version 3.4.2). FlashWeave version 0.19.2 was ran on Julia version 1.11.6.<br>All code used for microbial profiling and network construction and analysis is available in a GitHub repository (https://github.com/blacruz17/MHOmicrobiome) (DOI https://doi.org/10.5281/zenodo.19484217)

For manuscripts utilizing custom algorithms or software that are central to the research but not yet described in published literature, software must be made available to editors and reviewers. We strongly encourage code deposition in a community repository (e.g. GitHub). See the Nature Portfolio guidelines for submitting code & software for further information.

Data

Policy information about availability of data

All manuscripts must include a data availability statement. This statement should provide the following information, where applicable:

- Accession codes, unique identifiers, or web links for publicly available datasets
- A description of any restrictions on data availability
- For clinical datasets or third party data, please ensure that the statement adheres to our policy

AI4Food sequencing data are available at the European Nucleotide Archive with accession code PRJEB87701 [https://www.ebi.ac.uk/ena/browser/view/PRJEB87701], and patient metadata are provided in the project's GitHub repository [https://github.com/AI4Food/AI4FoodDB]. The processed sequencing data (taxonomy tables) as well as network files (edge lists) are available in a GitHub repository [https://github.com/blacruz17/MHOmicrobiome]. Sample metadata for the remaining studies can be accessed via curatedMetagenomicData or their source publications, cited in the main text. Supplementary Data 3 provides subject IDs, accession numbers, and phenotype classifications for individuals in each study. Sequencing data from the remaining studies used here are available in the ENA under project accession codes PRJEB7774 [https://www.ebi.ac.uk/ena/browser/view/PRJEB7774], PRJEB1786 [https://www.ebi.ac.uk/ena/browser/view/PRJEB1786], PRJEB41311 [https://www.ebi.ac.uk/ena/browser/view/PRJEB41311], PRJEB38742 [https://www.ebi.ac.uk/ena/browser/view/PRJEB38742], and PRJEB37249 [https://www.ebi.ac.uk/ena/browser/view/PRJEB37249].

Research involving human participants, their data, or biological material

Policy information about studies with human participants or human data. See also policy information about sex, gender (identity/presentation), and sexual orientation and race, ethnicity and racism.

Reporting on sex and gender	Information on AI4Food participants sex was self-reported. In the remaining studies, the information obtained from the metadata was collected.
Reporting on race, ethnicity, or other socially relevant groupings	Data on socially relevant variables such as race or ethnicity were neither collected nor considered in this research, where the focus was on gut microbiota patterns pertaining to metabolic health and obesity phenotypes.
Population characteristics	Patients were classified as overweight or obese based on WHO guidelines, while their metabolic health phenotypes were determined using BioShare-EU Healthy Obese Project criteria (van Vliet-Ostaptchouk et al. BMC Endocr. Disord. 2014). We have provided information on sex for all study groups (Table 2) and for all the studies retrieved (Supplementary Table 4).<br><br>All statistical analyses based on linear models have been adjusted for the effect of sex. For network inference, neighborhood selection with age and sex as covariates was performed using FlashWeave (Supplementary Methods).
Recruitment	For public data collection, we mined the database contained within the curatedMetagenomicData R package (version 3.8) for datasets with stool samples, with adult subjects that had not been treated with antibiotics recently, and where any of the variables of interest (described in Methods section) were available.<br>Patient recruitment in AI4Food was performed through the GENYAL Clinical Trials Platform mailing list and was complemented by a recruitment campaign via social media, the IMDEA Food Institute website, and the dissemination of information in nearby entities. Inclusion criteria: age 18 or older, body mass index between 25 and 35 kg/m2, ability to use required devices. Exclusion criteria: dementia, mental illness or decreased cognitive function, severe health conditions, pregnancy/breastfeeding, pharmacological treatment for weight loss. For more information access Lacruz-Pleguezuelos et al, Clinical Nutrition 2025 (https://doi.org/10.1016/j.clnu.2025.03.003).
Ethics oversight	The AI4Food study was approved by the Research Ethics Committee of the IMDEA Food Foundation (IMD PI-052; date of approval: 5th April 2022). Verbal and written informed consent were provided for all AI4Food participants. AI4Food participants received a small financial compensation for their time and inconvenience associated with study participation, in accordance with institutional guidelines and with approval from the Research Ethics Committee. Publicly available datasets were obtained from the R package curatedMetagenomicData and their source publications, where no personal data are included.

Note that full information on the approval of the study protocol must also be provided in the manuscript.

Field-specific reporting

Please select the one below that is the best fit for your research. If you are not sure, read the appropriate sections before making your selection.

☒ Life sciences ☐ Behavioural & social sciences ☐ Ecological, evolutionary & environmental sciences

For a reference copy of the document with all sections, see nature.com/documents/nr-reporting-summary-flat.pdf

Life sciences study design

All studies must disclose on these points even when the disclosure is negative.

Sample size	Sample sizes were not determined based on a priori statistical power calculations. Instead, all available samples meeting the inclusion criteria were included in the study. Our sample sizes were limited, particularly in the MHO case, by the lack of publicly available datasets with

	information regarding metabolic health. The smaller sample sizes in the MHNO and MHO groups have led to an imbalanced dataset, where it was difficult to obtain biomarker information using methods based on microbial abundances, leading us to choose a systems biology approach based on co-occurrence networks. Results were not affected by imbalances in sample sizes, as proven by our downsampling analyses. In any case, the sample size of our MHO group limits our statistical power, and thus results regarding this specific phenotype should be treated as exploratory. These limitations are explicitly included in the limitations section of our Discussion.
Data exclusions	We discarded curatedMetagenomicData studies performed on Asian, African, or non-Westernized populations to avoid geographic variability, as well as subjects affected by conditions outside of our scope (e.g., colorectal cancer or celiac disease) that could significantly impact gut microbiota composition.
Replication	Three replication strategies were followed. First, to assess whether the different sample sizes in each phenotype group could bias network properties, we performed subsampling analyses on MHNO, MUNO, and MUO groups. Second, to evaluate potential residual batch effects, we repeated these analyses on MHNO, MUNO and MUO samples from the largest study (MetaCardis). Third, an approach based on neighborhood selection with covariates was also employed to evaluate potential confounders (Supplementary Methods). All these strategies were successful. Results were also successfully replicated on longitudinal samples from the AI4Food cohort.
Randomization	No randomization was performed, as this was an observational study using pre-existing samples classified into predefined obesity and metabolic phenotypes (MHNO, MHO, MUNO, and MUO). Therefore, no experimental allocation of samples or subjects to groups took place. Potential confounding factors were addressed by applying consistent inclusion criteria across groups and, where applicable, by incorporating relevant covariates into the statistical analyses. Randomization was not considered applicable given the study design.
Blinding	Researchers were not blinded to group allocation during data collection or analysis. Samples were pre-classified into obesity and metabolic phenotypes (MHNO, MHO, MUNO, and MUO) based on established clinical criteria, which were integral to the study design and required for all downstream analyses. No experimental intervention or allocation of subjects to groups was performed. Blinding was therefore not considered relevant, as the study aimed to perform comparative analyses between predefined groups.

Behavioural & social sciences study design

All studies must disclose on these points even when the disclosure is negative.

Study description	*Briefly describe the study type including whether data are quantitative, qualitative, or mixed-methods (e.g. qualitative cross-sectional, quantitative experimental, mixed-methods case study).*
Research sample	*State the research sample (e.g. Harvard university undergraduates, villagers in rural India) and provide relevant demographic information (e.g. age, sex) and indicate whether the sample is representative. Provide a rationale for the study sample chosen. For studies involving existing datasets, please describe the dataset and source.*
Sampling strategy	*Describe the sampling procedure (e.g. random, snowball, stratified, convenience). Describe the statistical methods that were used to predetermine sample size OR if no sample-size calculation was performed, describe how sample sizes were chosen and provide a rationale for why these sample sizes are sufficient. For qualitative data, please indicate whether data saturation was considered, and what criteria were used to decide that no further sampling was needed.*
Data collection	*Provide details about the data collection procedure, including the instruments or devices used to record the data (e.g. pen and paper, computer, eye tracker, video or audio equipment) whether anyone was present besides the participant(s) and the researcher, and whether the researcher was blind to experimental condition and/or the study hypothesis during data collection.*
Timing	*Indicate the start and stop dates of data collection. If there is a gap between collection periods, state the dates for each sample cohort.*
Data exclusions	*If no data were excluded from the analyses, state so OR if data were excluded, provide the exact number of exclusions and the rationale behind them, indicating whether exclusion criteria were pre-established.*
Non-participation	*State how many participants dropped out/declined participation and the reason(s) given OR provide response rate OR state that no participants dropped out/declined participation.*
Randomization	*If participants were not allocated into experimental groups, state so OR describe how participants were allocated to groups, and if allocation was not random, describe how covariates were controlled.*

Ecological, evolutionary & environmental sciences study design

All studies must disclose on these points even when the disclosure is negative.

Study description	*Briefly describe the study. For quantitative data include treatment factors and interactions, design structure (e.g. factorial, nested, hierarchical), nature and number of experimental units and replicates.*
Research sample	*Describe the research sample (e.g. a group of tagged Passer domesticus, all Stenocereus thurberi within Organ Pipe Cactus National Monument), and provide a rationale for the sample choice. When relevant, describe the organism taxa, source, sex, age range and any manipulations. State what population the sample is meant to represent when applicable. For studies involving existing datasets, describe the data and its source.*
Sampling strategy	*Note the sampling procedure. Describe the statistical methods that were used to predetermine sample size OR if no sample-size calculation was performed, describe how sample sizes were chosen and provide a rationale for why these sample sizes are sufficient.*

Data collection | *Describe the data collection procedure, including who recorded the data and how.*

Timing and spatial scale | *Indicate the start and stop dates of data collection, noting the frequency and periodicity of sampling and providing a rationale for these choices. If there is a gap between collection periods, state the dates for each sample cohort. Specify the spatial scale from which the data are taken*

Data exclusions | *If no data were excluded from the analyses, state so OR if data were excluded, describe the exclusions and the rationale behind them, indicating whether exclusion criteria were pre-established.*

Reproducibility | *Describe the measures taken to verify the reproducibility of experimental findings. For each experiment, note whether any attempts to repeat the experiment failed OR state that all attempts to repeat the experiment were successful.*

Randomization | *Describe how samples/organisms/participants were allocated into groups. If allocation was not random, describe how covariates were controlled. If this is not relevant to your study, explain why.*

Blinding | *Describe the extent of blinding used during data acquisition and analysis. If blinding was not possible, describe why OR explain why blinding was not relevant to your study.*

Did the study involve field work? ☐ Yes ☐ No

Field work, collection and transport

Field conditions | *Describe the study conditions for field work, providing relevant parameters (e.g. temperature, rainfall).*

Location | *State the location of the sampling or experiment, providing relevant parameters (e.g. latitude and longitude, elevation, water depth).*

Access & import/export | *Describe the efforts you have made to access habitats and to collect and import/export your samples in a responsible manner and in compliance with local, national and international laws, noting any permits that were obtained (give the name of the issuing authority, the date of issue, and any identifying information).*

Disturbance | *Describe any disturbance caused by the study and how it was minimized.*

Reporting for specific materials, systems and methods

We require information from authors about some types of materials, experimental systems and methods used in many studies. Here, indicate whether each material, system or method listed is relevant to your study. If you are not sure if a list item applies to your research, read the appropriate section before selecting a response.

Materials & experimental systems

n/a	Involved in the study
☒	☐ Antibodies
☒	☐ Eukaryotic cell lines
☒	☐ Palaeontology and archaeology
☒	☐ Animals and other organisms
☐	☒ Clinical data
☒	☐ Dual use research of concern
☒	☐ Plants

Methods

n/a	Involved in the study
☒	☐ ChIP-seq
☒	☐ Flow cytometry
☒	☐ MRI-based neuroimaging

Antibodies

Antibodies used | *Describe all antibodies used in the study; as applicable, provide supplier name, catalog number, clone name, and lot number.*

Validation | *Describe the validation of each primary antibody for the species and application, noting any validation statements on the manufacturer's website, relevant citations, antibody profiles in online databases, or data provided in the manuscript.*

Eukaryotic cell lines

Policy information about cell lines and Sex and Gender in Research

Cell line source(s) | *State the source of each cell line used and the sex of all primary cell lines and cells derived from human participants or vertebrate models.*

Authentication | *Describe the authentication procedures for each cell line used OR declare that none of the cell lines used were authenticated.*

Mycoplasma contamination | *Confirm that all cell lines tested negative for mycoplasma contamination OR describe the results of the testing for mycoplasma contamination OR declare that the cell lines were not tested for mycoplasma contamination.*

Commonly misidentified lines (See ICLAC register) | *Name any commonly misidentified cell lines used in the study and provide a rationale for their use.*

Palaeontology and Archaeology

Specimen provenance | *Provide provenance information for specimens and describe permits that were obtained for the work (including the name of the issuing authority, the date of issue, and any identifying information). Permits should encompass collection and, where applicable, export.*

Specimen deposition | *Indicate where the specimens have been deposited to permit free access by other researchers.*

Dating methods | *If new dates are provided, describe how they were obtained (e.g. collection, storage, sample pretreatment and measurement), where they were obtained (i.e. lab name), the calibration program and the protocol for quality assurance OR state that no new dates are provided.*

☐ Tick this box to confirm that the raw and calibrated dates are available in the paper or in Supplementary Information.

Ethics oversight | *Identify the organization(s) that approved or provided guidance on the study protocol, OR state that no ethical approval or guidance was required and explain why not.*

Note that full information on the approval of the study protocol must also be provided in the manuscript.

Animals and other research organisms

Policy information about studies involving animals; ARRIVE guidelines recommended for reporting animal research, and Sex and Gender in Research

Laboratory animals | *For laboratory animals, report species, strain and age OR state that the study did not involve laboratory animals.*

Wild animals | *Provide details on animals observed in or captured in the field; report species and age where possible. Describe how animals were caught and transported and what happened to captive animals after the study (if killed, explain why and describe method; if released, say where and when) OR state that the study did not involve wild animals.*

Reporting on sex | *Indicate if findings apply to only one sex; describe whether sex was considered in study design, methods used for assigning sex. Provide data disaggregated for sex where this information has been collected in the source data as appropriate; provide overall numbers in this Reporting Summary. Please state if this information has not been collected. Report sex-based analyses where performed, justify reasons for lack of sex-based analysis.*

Field-collected samples | *For laboratory work with field-collected samples, describe all relevant parameters such as housing, maintenance, temperature, photoperiod and end-of-experiment protocol OR state that the study did not involve samples collected from the field.*

Ethics oversight | *Identify the organization(s) that approved or provided guidance on the study protocol, OR state that no ethical approval or guidance was required and explain why not.*

Note that full information on the approval of the study protocol must also be provided in the manuscript.

Clinical data

Policy information about clinical studies

All manuscripts should comply with the ICMJE guidelines for publication of clinical research and a completed CONSORT checklist must be included with all submissions.

Clinical trial registration | Publicly available data were obtained from the curatedMetagenomicData R package (version 3.8). Subject IDs employed are described in Supplementary Data 3. Source publications are cited in the main text. Regarding the AI4Food study, we provide the registered clinical trial identifier (ClinicalTrials.gov ID NCT05807243) for transparency and clarity. This trial included multiple participant populations. For the purposes of the present manuscript, we specifically analyzed a subset of approximately 100 samples from the overweight and obese population, which was the population of interest for our study objectives.

Study protocol | After merging the curatedMetagenomicData and AI4Food datasets, microbial species with an abundance above 0.01% in 10% of the samples were kept for further analyses. The MMUPHin R package (version 1.14) was used for batch effect correction caused by the study of origin while controlling for the effect of metabolic health and obesity. The effect of batch adjustment was evaluated based on the total variability in microbial profiles attributable to differences in the study of origin. This was done with a permutational multivariate analysis of variance (PERMANOVA) with 999 random permutations using the adonis2 function from the vegan R package (version 2.6-8). Further details are described in the Methods section.

Data collection | Publicly available data: we searched the data available within the curatedMetagenomicData R package (version 3.8). The source publications of each dataset were accessed to look for inclusion criteria and for further variables that might not have been included in curatedMetagenomicData. Subject IDs employed are described in Supplementary Data 3. Source publications are cited in the main

text. AI4Food project (publicly data available): we included 98 samples from the AI4Food project, carried out on a cohort of 100 obese and overweight subjects. For more information, see Lacruz-Pleguezuelos et al, Clinical Nutrition 2025 (https://doi.org/10.1016/j.clnu.2025.03.003) and Romero-Tapiador et al, Database 2023 (https://doi.org/10.1093/database/baad049)

Outcomes

We have described taxonomic and network-based measurements in the study cohort.
Metagenomic data exploration: Alpha diversity was estimated through the Chao1 index for richness and Shannon's and Simpson's indices for diversity using the alpha function from the microbiome R package (version 1.22). For beta diversity, the Aitchison distance was calculated. Differences between groups were evaluated based on PERMANOVA with 999 permutations. Differential abundance testing was performed with Analysis of Compositions of Microbiomes with Bias Correction 2 (ANCOM-BC2).
Network-based measurements: network order, size, edge density, number of connected components, degree, shortest path length, betweenness and closeness centrality, k-core distributions, keystone taxa, resistance to random and targeted attacks. Network analyses were done using the SpiecEasi R package (version 1.1.3), igraph R package (version 2.1.4), NetworkX Python library (version 3.4.2) and FlashWeave version 0.19.2 (ran on Julia version 1.11.6).

Dual use research of concern

Policy information about dual use research of concern

Hazards

Could the accidental, deliberate or reckless misuse of agents or technologies generated in the work, or the application of information presented in the manuscript, pose a threat to:

No	Yes	
☐	☐	Public health
☐	☐	National security
☐	☐	Crops and/or livestock
☐	☐	Ecosystems
☐	☐	Any other significant area

Experiments of concern

Does the work involve any of these experiments of concern:

No	Yes	
☐	☐	Demonstrate how to render a vaccine ineffective
☐	☐	Confer resistance to therapeutically useful antibiotics or antiviral agents
☐	☐	Enhance the virulence of a pathogen or render a nonpathogen virulent
☐	☐	Increase transmissibility of a pathogen
☐	☐	Alter the host range of a pathogen
☐	☐	Enable evasion of diagnostic/detection modalities
☐	☐	Enable the weaponization of a biological agent or toxin
☐	☐	Any other potentially harmful combination of experiments and agents

Plants

Seed stocks

n/a

Novel plant genotypes

n/a

Authentication

n/a

ChIP-seq

Data deposition

☐ Confirm that both raw and final processed data have been deposited in a public database such as GEO.

☐ Confirm that you have deposited or provided access to graph files (e.g. BED files) for the called peaks.

Data access links<br>*May remain private before publication.*	*For "Initial submission" or "Revised version" documents, provide reviewer access links. For your "Final submission" document, provide a link to the deposited data.*
Files in database submission	*Provide a list of all files available in the database submission.*
Genome browser session<br>(e.g. UCSC)	*Provide a link to an anonymized genome browser session for "Initial submission" and "Revised version" documents only, to enable peer review. Write "no longer applicable" for "Final submission" documents.*

Methodology

Replicates	*Describe the experimental replicates, specifying number, type and replicate agreement.*
Sequencing depth	*Describe the sequencing depth for each experiment, providing the total number of reads, uniquely mapped reads, length of reads and whether they were paired- or single-end.*
Antibodies	*Describe the antibodies used for the ChIP-seq experiments; as applicable, provide supplier name, catalog number, clone name, and lot number.*
Peak calling parameters	*Specify the command line program and parameters used for read mapping and peak calling, including the ChIP, control and index files used.*
Data quality	*Describe the methods used to ensure data quality in full detail, including how many peaks are at FDR 5% and above 5-fold enrichment.*
Software	*Describe the software used to collect and analyze the ChIP-seq data. For custom code that has been deposited into a community repository, provide accession details.*

Flow Cytometry

Plots

Confirm that:

☐ The axis labels state the marker and fluorochrome used (e.g. CD4-FITC).

☐ The axis scales are clearly visible. Include numbers along axes only for bottom left plot of group (a 'group' is an analysis of identical markers).

☐ All plots are contour plots with outliers or pseudocolor plots.

☐ A numerical value for number of cells or percentage (with statistics) is provided.

Methodology

Sample preparation	*Describe the sample preparation, detailing the biological source of the cells and any tissue processing steps used.*
Instrument	*Identify the instrument used for data collection, specifying make and model number.*
Software	*Describe the software used to collect and analyze the flow cytometry data. For custom code that has been deposited into a community repository, provide accession details.*
Cell population abundance	*Describe the abundance of the relevant cell populations within post-sort fractions, providing details on the purity of the samples and how it was determined.*
Gating strategy	*Describe the gating strategy used for all relevant experiments, specifying the preliminary FSC/SSC gates of the starting cell population, indicating where boundaries between "positive" and "negative" staining cell populations are defined.*

☐ Tick this box to confirm that a figure exemplifying the gating strategy is provided in the Supplementary Information.

Magnetic resonance imaging

Experimental design

Design type	*Indicate task or resting state; event-related or block design.*
Design specifications	*Specify the number of blocks, trials or experimental units per session and/or subject, and specify the length of each trial or block (if trials are blocked) and interval between trials.*

Behavioral performance measures

State number and/or type of variables recorded (e.g. correct button press, response time) and what statistics were used to establish that the subjects were performing the task as expected (e.g. mean, range, and/or standard deviation across subjects).

Acquisition

Imaging type(s)

Specify: functional, structural, diffusion, perfusion.

Field strength

Specify in Tesla

Sequence & imaging parameters

Specify the pulse sequence type (gradient echo, spin echo, etc.), imaging type (EPI, spiral, etc.), field of view, matrix size, slice thickness, orientation and TE/TR/flip angle.

Area of acquisition

State whether a whole brain scan was used OR define the area of acquisition, describing how the region was determined.

Diffusion MRI ☐ Used ☐ Not used

Preprocessing

Preprocessing software

Provide detail on software version and revision number and on specific parameters (model/functions, brain extraction, segmentation, smoothing kernel size, etc.).

Normalization

If data were normalized/standardized, describe the approach(es): specify linear or non-linear and define image types used for transformation OR indicate that data were not normalized and explain rationale for lack of normalization.

Normalization template

Describe the template used for normalization/transformation, specifying subject space or group standardized space (e.g. original Talairach, MNI305, ICBM152) OR indicate that the data were not normalized.

Noise and artifact removal

Describe your procedure(s) for artifact and structured noise removal, specifying motion parameters, tissue signals and physiological signals (heart rate, respiration).

Volume censoring

Define your software and/or method and criteria for volume censoring, and state the extent of such censoring.

Statistical modeling & inference

Model type and settings

Specify type (mass univariate, multivariate, RSA, predictive, etc.) and describe essential details of the model at the first and second levels (e.g. fixed, random or mixed effects; drift or auto-correlation).

Effect(s) tested

Define precise effect in terms of the task or stimulus conditions instead of psychological concepts and indicate whether ANOVA or factorial designs were used.

Specify type of analysis: ☐ Whole brain ☐ ROI-based ☐ Both

Statistic type for inference

(See Eklund et al. 2016)

Specify voxel-wise or cluster-wise and report all relevant parameters for cluster-wise methods.

Correction

Describe the type of correction and how it is obtained for multiple comparisons (e.g. FWE, FDR, permutation or Monte Carlo).

Models & analysis

n/a	Involved in the study
☐	☐ Functional and/or effective connectivity
☐	☐ Graph analysis
☐	☐ Multivariate modeling or predictive analysis

Functional and/or effective connectivity

Report the measures of dependence used and the model details (e.g. Pearson correlation, partial correlation, mutual information).

Graph analysis

Report the dependent variable and connectivity measure, specifying weighted graph or binarized graph, subject- or group-level, and the global and/or node summaries used (e.g. clustering coefficient, efficiency, etc.).

Multivariate modeling and predictive analysis

Specify independent variables, features extraction and dimension reduction, model, training and evaluation metrics.
